# Typhoid Fever in America

**Published:** 1900-03-17

**Authors:** 


### TYPHOID FEVER IN AMERICA.

Again it seems that Philadelphia will have to pay the penalty for its remissness in the matter of providing its citizens with reasonably pure drinking water. For some time past, we are told, the drinking water in that city has been filthy and most unattractive for either bathing or drinking; but a short time ago some mischievous boys interfered with an intercepting sewer in such a way that sewage overflowed into the Schuylkill not far from a pumping station, with the apparent result of rapidly increasing the prevalence of typhoid fever, the number of cases reported to the Philadelphia Bureau during successive weeks from January 20th to February 17th being as follows: 35, 44, 39, 56, and 76 cases. For some time back Philadelphia has had under consideration the report of a commission of experts on water purification, which it has adopted, and for carrying out the recommendation of which a large sum of money has been voted, but nothing is done; and we may safely opine that so long as the present condition of affairs continues, and the custom of using unfiltered river water for drinking purposes, so common in American, persists, these outbreaks of typhoid fever will continue to recur.

